# Population dynamics and associated factors of cereal aphids and armyworms under global change

**DOI:** 10.1038/srep18801

**Published:** 2015-12-22

**Authors:** Leyun Wang, Cang Hui, Hardev S. Sandhu, Zhihong Li, Zihua Zhao

**Affiliations:** 1 Department of Entomology, College of Plant Protection, China Agricultural University, Beijing, 100193, China; 2 Centre for Invasion Biology, Department of Mathematical Sciences, Stellenbosch University, Matieland 7602, South Africa; 3 Mathematical and Physical Biosciences, African Institute for Mathematical Sciences, Muizenberg 7945, South Africa; 4 Everglades Research and Education Center, Institute of Food and Agricultural Sciences, University of Florida, Belle Glade, USA

## Abstract

Studying the impacts of global change, which comprises largely climate and landscape changes, on agricultural pests is crucial for developing sustainable pest management. This research is focused on understanding the factors associated with population dynamics of cereal aphids and armyworms feeding on wheat in Henan province in China from 1987 to 2010. Association between changes in climate (temperature, precipitation, and relative humidity) and agricultural characteristics (wheat proportion, crop diversity, fertilizer input, and wheat yield per unit area) and damage from cereal aphids and armyworms were examined. Cereal aphid damage has been rising, while armyworm damage had no obvious trends, but with strong year-to-year fluctuations. The analysis indicates that the factors most strongly associated with the population dynamics of cereal aphids are fertilizer input and mean temperature in February, while the population dynamics of armyworms is significantly related to precipitation in May. By comparing the characteristics of these two agricultural pests, we identify possible reasons for the disparity between their associated factors, which are related to the differences in their foraging behaviour, host range, migration capacity, and life history. These results may contribute to developing ecologically based pest management for cereal aphids and armyworms under global change.

Global change comprises changes in climate and land use driven by natural and anthropogenic processes^1^. Modeling and understanding the associated factors with pest population dynamics under global change are great challenges for developing ecologically based pest management. On the one hand, climate changes could affect population dynamics directly or indirectly through shifting survival, behaviour, and life cycles of insects^2^. Increasing temperature could facilitate physiology and reproduction of insects at an individual level^3^. However, whether climate changes could affect pest damage and population dynamics at a community level is still largely unknown. On the other hand, agricultural landscapes have also been changed greatly due to the shift of crop arrangement and land cover, which may drastically influence population dynamics of many pest species in the past decades^4^. Cropland expansion, which has led to the fragmentation of natural habitats and loss of biodiversity, and the intensification of agriculture (intensive use of high-yielding crop varieties, fertilizers, pesticides, and irrigation), has also caused degeneration of ecosystem functions and ecosystem services such as biological control^5^,^6^.

Many researchers have studied the impacts of climatic conditions on population dynamics and distribution ranges of insects (e.g.^3^,^7^,^8^,^9^,^10^,^11^,^12^). Increasing temperature may positively affect development, longevity, and fecundity of *Tetraneura nigriabdominalis* (Hemiptera: Aphididae) within a certain range^7^. An increase in temperature of merely 2 °C could increase the number of generations of aphids per year from 18 to 23 and result in a larger population size^3^. Moisture factors such as precipitation and humidity may also influence the aphid population diversely in different time and space under multiple conditions^8^,^9^. Studies on armyworms (Lepidoptera: Noctuidae) have demonstrated that the integrated effects of temperature and moisture are significant on their vital rates, developmental time and fecundity^10^. In Sharma *et al.*’s experiment^11^, rainfall, maximum, and minimum relative humidity were positively associated with oriental armyworm moth catches nearly one month after the larval peak. A study on a serious outbreak of the 3rd-generation armyworm larvae concluded that low temperature and frequent rainfall could have created favorable conditions for the breeding of the 2nd-generation adults and suitable hosts for the 3rd-generation larvae^12^.

Landscape changes, which shape the habitat structure, materials, and biotic interactions in agroecosystems, are also important factors driving pest population dynamics. Examining their impacts on pests or natural enemies and understanding their mechanisms may help to design pest management strategies at landscape scales. Generally, plant diversity may affect activity and diversity of insects. Aphid populations may be suppressed with habitat complexity both at the local scale (plant diversity) and at the landscape scale (landscape heterogeneity)^13^,^14^. Increasing fertilizer input has a major influence on the material flow in the agroecosystem^15^. The application of nitrogen fertilizer may increase natural populations of some cereal aphids^16^. Additionally, aphids can become more abundant in conventionally fertilized barley compared to organically fertilized barley because of changes in plant morphology or the temporal availability of nutrients^17^. Besides, large yield of crop resulted mainly from increasing fertilizer input could also support larger populations of pests.

Many researchers have conducted experiments to investigate the key factors driving population dynamics of various pests and their natural enemies at a local scale, but studies have rarely been done at a large scale such as a province. With comprehensive data of pests, climate, and landscape in Henan, an agriculture dominated province with the highest wheat yield in China for many years, here we investigate factors associated with the population dynamics of cereal aphids and armyworms feeding on wheat under global change. Cereal aphids are major wheat sap sucking pests throughout the world, completing about 10 to 20 generations per year and experiencing strong year-to-year fluctuations^18^. Armyworms are seasonal and long distance migratory chewing pests, completing about 3 to 4 generations per year. Both the cereal aphid and the armyworm have caused severe damage to crops in China^19^. Together with previous small-scale studies, results from this study may provide a broader view on the population dynamics of wheat pests at a large scale.

## Results

### Climate and landscape change

As expected, there were year-to-year changes in climate in Henan province from 1987 to 2010. Temperature during the wheat growing season rose with enhancing fluctuations. Temperature during the overwintering period experienced increasing fluctuations without any obvious trend. Precipitation and relative humidity (RH) during the wheat growing season and the overwintering period fluctuated strongly between years without a clear trend. Years with extreme precipitation (1991, 1998, 2001, and 2002) also had extreme RH during the wheat growing season (Supplementary Dataset S1).

Landscape changes were remarkable from 1987 to 2010. The proportion of wheat planting area to total arable area (wheat proportion) was increasing in the first 11 years and then decreasing with fluctuations. Crop diversity, fertilizer input, and wheat yield per unit area were all increasing during this period. Fertilizer input in 2010 was about 5 times as high as in 1987, and wheat yield per unit area in 2010 was about 1.7 times as high as in 1987 (Supplementary Dataset S2).

### Cereal Aphid

**Figure 1:**
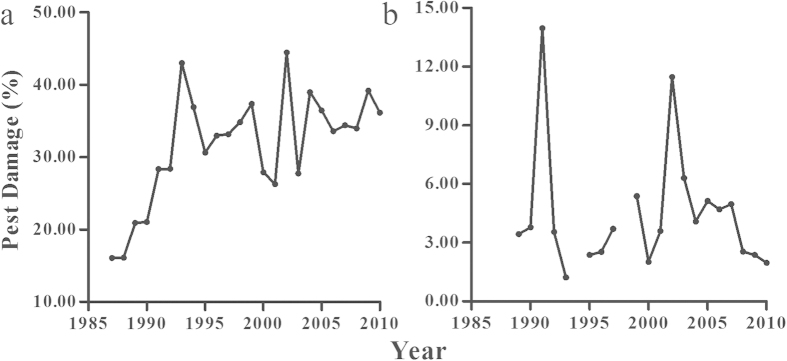
Pest damage of cereal aphids and armyworms in Henan province during 1987 to 2010. (**a**): cereal aphid, (**b**): armyworm.

The pest damage (PD) of cereal aphids rose with fluctuations from 1987 to 2010 (Fig. 1a; Supplementary Dataset S3). Selected models comprised fertilizer input, mean temperature in February, precipitation in April, and wheat yield per unit area (Table 1), of which only fertilizer input and mean temperature in February were significant in determining the PD of cereal aphids. As such, fertilizer input and temperature in February could have strong effects on the population dynamics of cereal aphids. Steadily increasing fertilizer input might contribute the most to the rise of cereal aphids. The amount of nitrogen fertilizer used in agriculture was found aligning with the trend of total fertilizer applied in agroecosystems, which could be the underlying associated factor behind the rise of cereal aphids. Winter wheat and cereal aphid are both in critical development stages in February in the region. Better heat conditions in February could proliferate the population growth of cereal aphids both directly and indirectly.

### Armyworm

The PD of armyworm had no obvious trend from 1989 to 2010, and experienced strong year-to-year fluctuations (Fig. 1b; Supplementary Dataset S3). The best model (model a1) for predicting the armyworm PD, with the lowest AICc score in candidate models, included the significant effects of precipitation in May, whereas a marginally supporting model a4 (∆AICc = 7.16) had a significant predictor from the precipitation during the growing season (Table 2). As precipitation during the growing season includes precipitation in May, they were used in separate models (Supplementary Dataset S4). Precipitation during the growing season or in May, alone, may be the key factor impacting the population dynamics of armyworms. Notable contributions from extremely large amount of precipitation during the growing season or in May to severe outbreaks of armyworms were detected from data graphs and historical reports; most of the drought years coincided with the smallest armyworm outbreaks. These findings further validated the critical role of precipitation during the growing season or in May in driving the population dynamics of armyworms.

## Discussion

There were noticeable changes in both climate and landscape factors from 1987 to 2010 in Henan province, while pest damage of cereal aphids and armyworm fluctuated strongly between years. This study was set up to explore the associated factors with population dynamics of each pest by examining associations between candidate climate and landscape variables and pest damage. We found that fertilizer input and mean temperature in February were significantly correlated with pest damage of cereal aphids. The precipitation in May or during the wheat growing season could significantly drive the population dynamics of armyworms. The results are in general agreement with many previous studies at individual and population levels, which together could help us to understand the underlying mechanisms of these key factors in influencing some agricultural pest populations.

The significant positive roles of fertilizer input and mean temperature in February in aggravating the damage from cereal aphids have been reported by many researches (e.g.^3^,^17^,^20^,^21^). Increasing fertilizer input has a major influence on the material flow through the agroecosystem^15^, and nitrogen application specially improves wheat growth and consequently supports larger cereal aphid populations^20^,^21^. Intensified use of nitrogen fertilizer may enlarge leaf size^22^, tender stalks^23^, and alter colour of wheats^17^, therefore enhancing the feeding preference of cereal aphids^24^. The considerably positive relationship between total wheat yield and fertilizer input implies a great improvement in wheat nutrition status that could indirectly promote the development, growth, and reproduction of cereal aphids^25^,^26^. In February, wheat in Henan is returning green, while cereal aphids are getting active after overwintering^27^. Higher temperature could facilitate root growth, leaf production, and tillering that could result in higher yield in the end. Cereal aphids may benefit from the improved nutrition of wheats even throughout the entire growing season. Higher temperature could increase the overwintering survival rate and cause phenological advances in the waking of cereal aphids, and better heat condition could also contribute to their population growth^28^,^29^.

The best model for armyworm featured a significant positive impact from the precipitation in May (the last month during the growing season) on pest damage, while another supporting model also indicated the significant effects of precipitation during the growing season. As precipitation within a year or a season often has a similar trend, we looked more into the effects of precipitation during the entire growing season. Armyworms are migratory pests flying to Henan province from the south to produce the first generation larvae in late March. They then forage on crops and reach their damage peak. Finally, they emerge and continue migrating to the further north in late May and early June^30^. Wheat growth during the growing season (March, April, and May) provides armyworms with abundant food resources. Rainfall may influence the microclimate especially the humidity substantially, and the humidity could affect armyworms directly in multiple ways^10^. The effects of rainfall and humidity on armyworm individuals and populations have been reported in many researches (e.g.^11^,^12^,^31^,^32^,^33^,^34^). In Jin’s experiments, fecundity, egg development, hatching rate, larval survival, and larval development were shown to be positively impacted by high RH^31^. Low humidity makes the egg shell a more difficult obstacle for larvae to chew their way out. Higher humidity in air or soil directly brings about higher survival rates of the 1st, 2nd, and 4th instar larvae^10^. Therefore, humidity could be an important factor affecting the formation of the initial population. Low humidity also reduces vigor of larvae and hence reduces their ability to cause damage. The duration of larval stage is inversely correlated to humidity, so larvae can reach their foraging peak earlier with higher humidity^10^. Wheat enters harvest time in May, while armyworms are in their gluttony state (5^th^ and 6^th^ instar larvae). This synchrony may explain why in the best model the precipitation in May is the key variable to determine the PD of armyworms during one growing season.

It was found that most of the extreme values in precipitation data of growing seasons were corresponding to most of the extreme RH values such as in 1991, 1998, 2001, and 2002, when there were extreme outbreaks or suppression of armyworms. Besides, RH was strongly associated with precipitation in May and some other months. However, we did not include RH in May in our supporting models to avoid collinearity between explanatory variables.

There were two serious outbreaks of armyworms in 1991 and 2002, and the precipitation in May and during the growing season were much higher in these two years. It is likely that massive rainfall has driven these serious outbreaks. However, there were also some years with relatively large amount of precipitation in May or during the growing season yet without any serious armyworm outbreaks. It may be because heavy downpour could wash the armyworms off the plants, causing or bodily injuries^10^. Moreover, the population of armyworms may be affected by several different factors that might not have been considered in our research. Some serious outbreaks of armyworms in Asia were attributed to drought after rainfall, floods, and humidity^11^. These cases demonstrate that the factors regulating armyworm population dynamics are complicated, but also support the results of our research that precipitation is a crucial factor.

In Henan province, cereal aphids and armyworms co-occur on wheat during about the same period, but the variables related to their population dynamics are completely different. There are some distinctions in their characteristics that may explain the differences. First, their foraging behaviours are different. Cereal aphids are sucking pests, while armyworms are chewing pests. Cereal aphids are phloem feeders and deplete the plant’s food reserves by imbibing large quantities of plant sap for nitrogen with their piercing-sucking mouthparts^21^,^28^. Nitrogen is in the highest demand of cereal aphids that will even excrete surplus carbohydrates for assimilating enough nitrogen. Nitrogen rich food supply can be a key factor supporting a larger population of cereal aphids. Furthermore, wheat may become more susceptible to these sucking pests due to higher nitrogen input^17^,^22^,^23^. Armyworm larvae have powerful chewing mouthparts that can cut the stalks, ears, and eat up the leaves^35^. More susceptible organs or tissues may not attract armyworms since these may never be limitations to them. This provides us a possible explanation for different impacts of fertilizer input on cereal aphids and armyworms.

Second, their host ranges are also different. Cereal aphids are oligophagous i.e. they mainly feed on cereals. Armyworms are polyphagous pests that forage on cereals, rice, millet, maize, cotton, beans, vegetables and more. In general, polyphagous insects have more flexible food resources than oligophagous insects. Their host ranges give us one other clue about different impacts of fertilizer input on their population dynamics. The wider range of hosts indicates that armyworms may be less fastidious than cereal aphids; as such, the changes in fertilizer input which could affect the consumption preferences of pests may be more critical to cereal aphids than armyworms.

Third, their migration capacities are different. Armyworms are strongly migratory and can spread throughout China within half a year. To remain active, armyworms can migrate to southern provinces without overwintering^36^. Compared to armyworms, the spatial distribution of cereal aphids is much more limited^37^,^38^. As a result, the temperature during the overwintering period at one location could strongly affect the population dynamics of cereal aphids. This may explain why February temperature in Henan affects them differently.

Fourth, their life histories also differ. In Henan province during one growing season, cereal aphid could complete over 6 generations^39^, while armyworms only have about 1 generation on wheat^30^. According to the previous discussion, the effects of precipitation on mortality and fecundity are essentially on the establishment of initial populations in each generation. This could make a huge difference regarding the population size for species with few generations per year. However, the population of cereal aphids can grow exponentially over 6 generations in Henan province during one growing season of wheat^39^. The effect of density-dependence between generations on the population size may be overshadowed by the rapid growth of population, and this may account for the different impacts of weather conditions on the population dynamics of these two pests during the wheat growing season.

## Methods

### Study Region and Species

The study region was Henan province (N: 31° 23′ − 36° 22′, E: 110° 21′ − 116° 39’, 167,000 km^2^) in North China. Henan province is traversed by the Yellow River that has partially led to the formation of North China Plain where Henan province is located (55.7% land of Henan province is plain). Henan province has a temperate monsoon climate with four distinct seasons, and it rains the most in summer. The mean temperature is 15.7 °C to 12.1 °C from south to north. The annual precipitation is 532.5 mm to 1380.6 mm. About 50% area of Henan province is arable land. The main crops are wheat (70% of arable land area), maize (31% of arable land area), and cotton (11% of arable land area, averaged data from 1987 to 2010). The climate and soil conditions in Henan province are suitable for winter wheat (the study crop in this research) planting. The wheat yield of Henan province has always been the highest in China.

Two types of wheat pest including cereal aphids and armyworms were selected in this study. *Sitobion avenae* Fabricius, *Schizaphis graminum* Rondani, and *Rhopalosiphum padi* Linn. are dominant cereal aphid species in wheat fields in Henan province. Cereal aphids are major wheat sap sucking pests throughout the world. Their population often experiences strong year-to-year fluctuations^18^. Cereal aphids complete about 10 to 20 generations per year. Armyworms comprise *Mythimna separata* Walker, *Leucania separata* Walker, and other armyworm species. Armyworms are seasonal and long distance migratory chewing pests. They cause severe damage to various agricultural crops in China, other parts of Asia, and Australia^19^. Armyworms complete about 3 to 4 generations per year.

### Data Collection

The data on annual outbreaks of cereal aphids (n = 24) and armyworms (n = 20) from 1987 to 2010 were collected by National Agricultural Technology Extension Service Center of China (missing data were excluded from the analysis; Supplementary Dataset S3). For each pest, the outbreak area was estimated and summed across 3 settled times throughout the wheat growing season per year (March to May in northern China) by using a standard method: outbreak area was only counted when the outbreak and damage levels of these pests exceeded their economic thresholds. The economic thresholds are 500 individuals/100 straws for cereal aphids and 67.5 individuals/100 straws for armyworms. A population density above the economic threshold is a signal of colonization success of the pests. Furthermore, higher insect abundance has been proven to be associated with wider distribution^40^. The population size of pests over economic threshold is regarded as causing considerable damage, although pests do occur in areas at a level below the economic threshold. At the provincial scale, we used the proportion of outbreak area above the economic threshold in total wheat area as an indicator of pest damage and pest population size (e.g.^41^,^42^,^43^,^44^). It is worth noting that the cereal aphids and armyworms studied here were only referred to those feeding on the wheat crop.

There were three climate factors: temperature, precipitation and humidity considered in this study (Supplementary Dataset S1). These data were obtained from China Meteorological Data Sharing Service System, China Meteorological Administration (http://cdc.nmic.cn/home.do/). The climate data from November and December of each year were used in analyzing the associated factors for next year’s pest population. The mean climate values of the whole province were calculated from three sites (Station No. 57083, 57297, and 57290) nearly equidistantly located from south to north on the medial axis along the longitude line in Henan province. In Henan province, the overwintering period is from December to February, and the wheat growing season from March to May.

The monthly mean temperature (T, °C) during 1986 to 2010 was used in the analysis. The mean temperature of the three months during the overwintering period (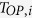) and the mean temperature of the three months during the wheat growing season (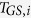) in year *i* were calculated as


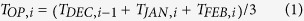



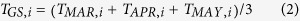


where 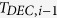 is the mean temperature in December in year *i*-1, and *T*_*JAN.i*_, *T*_*FEB.i*_, *T*_*MAR.i*_, *T*_*APR.i*_, and *T*_*MAY.i*_ are the mean temperatures in January, February, March, April, and May in year *i*, respectively.

The monthly precipitation (P, mm) during 1986 to 2010 was used in the analysis. Besides the direct impact of precipitation on pests, it may also make a difference to the farmland water supply and microclimate. The cumulative precipitation of the three months during the overwintering period (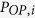) and the cumulative precipitation of the three months during the wheat growing season (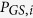) in year *i* were calculated as


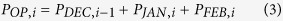



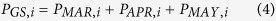


where 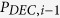 is the precipitation in December in year *i*-1, and 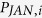,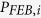, 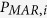, 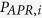 and 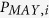
are the precipitations in January, February, March, April, and May in year *i*, respectively.

The mean relative humidity (RH, %) of each month during 1986 to 2010 was also used in the analysis. The mean *RH* of the three months during the overwintering period (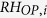) and the mean *RH* of the three months during the wheat growing season (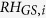) in year *i* were calculated as


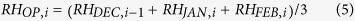



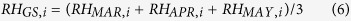


where 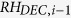 is the mean RH in December in year *i*-1, and 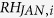, 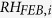, 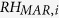, 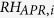 and 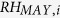 are the mean RHs in January, February, March, April, and May in year *i*, respectively.

In this study, we also included annual agricultural variables at the landscape scale, including wheat proportion, crop diversity, fertilizer input, and yield of wheat per unit area. These landscape data were calculated based on the raw data documented in *China Agriculture Year Book*^45^ (Supplementary Dataset S2).

The ratio of wheat planting area to total arable area is an indicator of wheat proportion (WP). Changes in wheat proportion can be a result from the choice of planting crops. This can modify the landscape structure in the agroecosystem. The *WP* for year *i* was calculated as


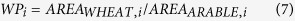


where 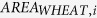 is the wheat planting area in year *i* and 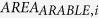 the total arable area in year *i*.

We used the Shannon-Wiener Index to capture the compositional diversity of the agroecosystem at the province level. The crop diversity (CD) was calculated from the planting areas of different crops, including areas for grain, vegetable, sugar, tobacco, fruit, oil, and medicinal plants, which together occupy most croplands in Henan province, as the following


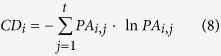


where *s* is the total number of crop types in Henan province, and 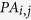 is the proportion of planting area for crop type *j* to the total planting area in year *i*.

Data on fertilizer input (FI, kg) in each year were available only for the entire agroecosystem in Henan province, so we could only assess the primary trend of fertilizer input to the entire system. However, wheat has been consistently taking up about 70% of the cropland in Henan province, so it is sufficient for us to assume that the fertilizer input to wheat fields had the same changes^46^.

The yield per unit area could also be an effective indicator of the nutrition status of wheat. Yield per unit area of wheat (YPU, kg/ha) for year *i* was calculated as


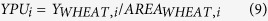


where 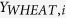 is the total yield of wheat in year *i*, and 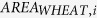 the wheat planting area of year *i*.

### Statistics

We used the percentage of the pest outbreak area in the total wheat planting area as the indicator for pest damage (PD, %), as it was calculated based on both the severity and the quantity of the damage by pests (i.e. the proportion of areas with the pest density above the economic threshold). Both the damage to wheats and the population of cereal aphids and armyworms could be captured by the index of PD^46^. The *PD* for pest *k* in year *i* was calculated as


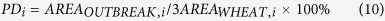


where 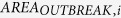 is the outbreak area of pest *k* in year *i* and 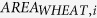 is the wheat planting area of year *i*. We used the total affected area from three surveys during one wheat growing season as 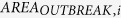; consequently it was divided by three in the above formula.

In summary, we tested multiple factors that could have an impact on the PD, including temperature, precipitation, RH, wheat proportion, crop diversity, fertilizer input, and wheat yield per unit area. With these explanatory variables, we tested multiple candidate models based on literature, experience and observations in the field. All multiple linear regression models built were assessed whether the assumptions of the modeling procedure were met. The distribution of full-model residuals was diagnosed by the Shapiro-Wilk test, and the residuals showed to be normally distributed (cereal aphids: W = 0.98121, p-value = 0.9171; armyworms: W = 0.96883, p-value = 0.73). We also examined the variables in each candidate model to avoid collinearity between variables (Supplementary Dataset S4). No temporal autocorrelation of pest damage of cereal aphids or armyworms was detected during 1987 to 2010^48^. Model fitting was performed using generalized linear models (GLM) in the statistical program R Version 3.2.1 (R Core Team, Austria). AIC-based model selection was conducted for different candidate models to select the best model and supporting models for explaining the PD of cereal aphids and armyworms^49^,^50^. We presented ∆AICc and r^2^ for selected models in Table 1 for cereal aphids and Table 2 for armyworms^51^. Across all selected models, we examined the significance of each explanatory variable through two-tailed t-tests for evaluating its role in determining the response variable^52^.

## Additional Information

**How to cite this article**: Wang, L. *et al.* Population dynamics and associated factors of cereal aphids and armyworms under global change. *Sci. Rep.* **5**, 18801; doi: 10.1038/srep18801 (2015).

## Supplementary Material

Supplementary Dataset S1

Supplementary Dataset S2

Supplementary Dataset S3

Supplementary Dataset S4

## Figures and Tables

**Table 1:** Comparison of selected models for explaining the pest damage of cereal aphids in Henan province from 1987 to 2010.

Model	Equation	df	∆AICc	r^2^
c1	12.060 + 126.400FI* + 0.172 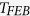 ** + 0.00755 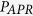	5	0.00	0.55
c2	15.001 + 168.748FI** + 0.144 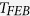 *	4	0.83	0.50
c3	24.896 + 331.414*FI *+ 0.168 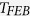 ** + 0.00722 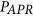 −55.099*YPU*	6	1.55	0.57
c4	19.176 + 209.765FI***	3	3.99	0.38

Note: *FI* represents fertilizer input; 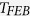 mean temperature in February; 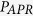 precipitation in April; *YPU* the yield of wheat per unit area.

Significance of variables is indicated as follows: *P < 0.05, **P < 0.01, ***P < 0.001.

**Table 2:** Comparison of selected models for explaining the pest damage of armyworms in Henan province from 1989 to 2010.

Model	Equation	df	∆AICc	r^2^
a1	−0.468 + 0.006 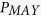 ***	3	0.00	0.48
a2	−0.014 + 0.006 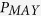 ***−0.001 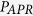	4	2.85	0.45
a3	−0.105 + 0.006 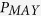 **−0.001 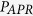 −0.001 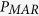	5	6.38	0.42
a4	−0.286 + 0.002 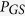 *	3	7.16	0.25

Note: *P*_*MAY*_ represents precipitation in May; *P*_*APR*_ precipitation in April, *P*_*MAR*_ precipitation in March, *P*_*GAS*_ precipitation during the entire growing season.

Significance of variables is indicated as follows: *P < 0.05, **P < 0.01, ***P < 0.001.
